# SUMO: regulating the regulator

**DOI:** 10.1186/1747-1028-1-13

**Published:** 2006-06-29

**Authors:** Guillaume Bossis, Frauke Melchior

**Affiliations:** 1 Institut de Génétique Moléculaire de Montpellier, CNRS, 1919 route de Mende, 34293 - Montpellier Cedex 05, France; 2 Dept. of Biochemie I, University Goettingen, Humboldt Allee 23, 37073 Goettingen, Germany

## Abstract

Post-translational modifiers of the SUMO (Small Ubiquitin-related Modifier) family have emerged as key regulators of protein function and fate. While the past few years have seen an enormous increase in knowledge on SUMO enzymes, substrates, and consequences of modification, regulation of SUMO conjugation is far from being understood. This brief review will provide an overview on recent advances concerning (i) the interplay between sumoylation and other post-translational modifications at the level of individual targets and (ii) global regulation of SUMO conjugation and deconjugation.

## The many roles of sumoylation

Sumoylation, which consists in the covalent and reversible conjugation of Small Ubiquitin-related Modifiers (SUMO-1, 2 and 3 in mammals) to target proteins, is an essential cellular process from yeasts to mammals. In *S. cerevisiae*, disruption of the SUMO pathway leads to a G2/M cell cycle arrest [1]; In mouse, it leads to embryonic death at early stages [2].

The number of known SUMO targets is growing exponentially, and it seems likely that this modification is as common as phosphorylation to regulate biological processes [3]. Most SUMO targets were initially found in the nucleus, but it is now clear that sumoylation also regulates cytoplasmic-, and even plasma membrane associated proteins. Modification has been linked to pathways as diverse as intracellular trafficking, cell cycle, DNA repair and replication, RNA metabolism and cell signalling (for detailed description of sumoylation functions see [4]). At a molecular level, sumoylation alters protein functions by masking and/or adding interaction surfaces, or by inducing conformational changes that result in altered interactions (for detailed examples see [5]). As a consequence, a wide variety of downstream consequences have been observed, including changes in localisation, enzymatic activity, or stability.

Among the many known targets of sumoylation, a large number are regulators of gene expression, in particular transcription factors, co-activators or repressors. Here, the emerging picture is that sumoylation essentially results in down-regulation of gene expression. Albeit the molecular mechanisms underlying this repression by SUMO are still ill defined, they seem to involve SUMO-dependent recruitment of transcriptional repressors such as HDACs directly to promoters [6].

## The SUMO conjugation and deconjugation pathway

**Figure 1:**
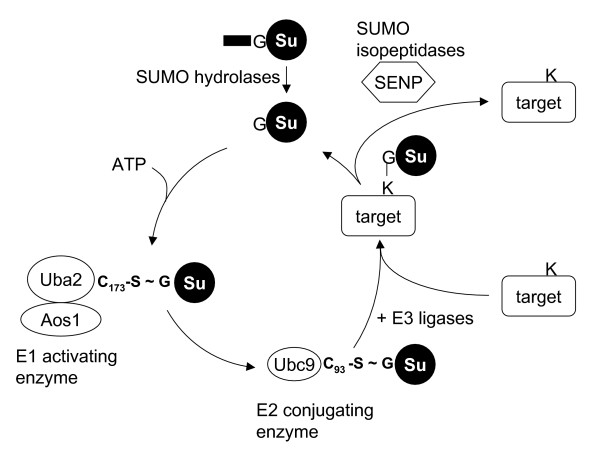
**The SUMO pathway**. All SUMO isoforms are synthetized as a precursor, containing a C-terminal extension, which is cleaved by specific hydrolases. Mature SUMO is then activated by formation of a thioester bond between its C-terminal glycine and the catalytic cysteine of the Uba2 subunit from the E1 activating enzyme (Aos1/Uba2). This step requires ATP hydrolysis. SUMO is then tranfered to the catalytic cysteine of the E2 activating enzyme Ubc9. The last step of the conjugating cascade consists in the transfer of SUMO from Ubc9 to the ε-NH2 group of a lysine side chain (isopeptide bond formation). Efficient modification usually requires E3 ligases. SUMOylation is a reversible and highly dynamic modification due to the presence of specific cysteine proteases of the Ulp/Senp family that cleave the isopeptide bond.

All SUMO isoforms are conjugated via a conserved enzymatic cascade that resembles that of ubiquitin conjugation (Figure 1). SUMO is first activated by formation of a thioester bond between its C-terminal glycine and the catalytic cysteine of the heterodimeric E1 activating enzyme (Aos1/Uba2, also named SAE1/SAE2). This step requires ATP hydrolysis. SUMO is then transferred to the catalytic cysteine of the single E2 conjugating enzyme Ubc9. The last step consists in the transfer of SUMO from the E2 to the ε-amino group of a lysine side chain on the substrate, which results in isopeptide bond formation. For this, Ubc9 needs to recognize a specific acceptor site in the target. Many – but not all – targets contain the so-called SUMO consensus motif, ψKxE, where Ψ is a large hydrophobic residue and K the acceptor lysine. The interaction between Ubc9 and most targets is not stable enough for efficient transfer, and therefore requires additional proteins, the so-called E3 ligases. Currently known ligases include PIAS (Protein inhibitor of activated STAT) proteins, the nucleoporin RanBP2/Nup358 and the polycomb protein Pc2 [7,5,4].

SUMO-target conjugates are susceptible to cleavage by specific cysteine proteases (SUMO isopeptidases) [7]. In yeast, two isopeptidases, Ulp1 and Ulp2, are responsible for SUMO deconjugation. Homologs of the Ulp family were identified by sequence homology and referred to as SENP proteins (1 to 8). SUMO isopeptidase activity was confirmed for SENP-1 [8], SENP-2 [9-11], SENP-3 [12,13], SENP5 [13] and SENP6 [14]. SENP-8, however, turned out to be a NEDD8 specific isopeptidase [15-17]. Whether other types of SUMO isopeptidases exist is still not clear. All SENPs share a conserved catalytic domain but have distinct N-terminal extensions, which might be responsible for their different intracellular localisations [7].

The interplay between modifying enzymes and isopeptidases is responsible, at least in part, for the late discovery of SUMO: with rare exceptions such as RanGAP1, the first protein for which sumoylation was shown, most SUMO targets are modified at very low steady state levels in vivo. Moreover, upon cell lysis most sumoylated proteins are highly susceptible to demodification by isopeptidases. Dynamic sumoylation/desumoylation cycles may also be responsible for a frequently observed conundrum: while only a small fraction of a given target is sumoylated at steady state, their corresponding SUMOylation-deficient mutants can have striking effects [4].

## Regulation of sumoylation at the substrate level

While selected SUMO targets are modified constitutively, many proteins are sumoylated in a temporally or spatially regulated fashion. Examples for target specific regulation are mitotic modification of yeast septins [18] and topoisomerase II [19], PML demodification [20] in mitosis, *S. c*. PCNA sumoylation in S-phase [21,22], stress induced sumoylation of heat shock factor I [23], or circadian cycle dependant sumoylation of BMAL1 [24]. While only fragmented knowledge concerning this regulation is available, it is already clear that there are several distinct mechanisms to enhance or inhibit sumoylation of individual target proteins.

**Figure 2:**
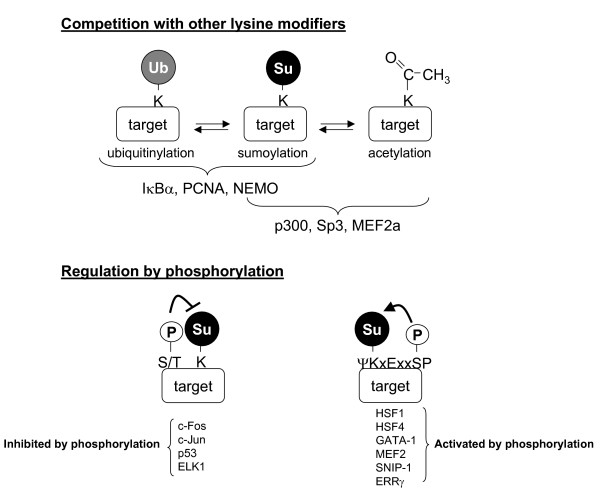
**Regulation of SUMOylation through target modification**. Distinct post-translational modifications of the target protein can affect its sumoylation. A) Competition with other lysine-directed modifications such as ubiquitinylation of acetylation abolishes sumoylation. B) Phosphorylation can act both as a positive and a negative regulator of sumoylation. Enhanced sumoylation has been observed, e.g., when the phosphorylated residue is part of an extended SUMO acceptor site, the PDSM (Phosphorylation Dependant SUMO Motif; with the sequence motif ΨKxExxSP [30]).

One obvious way to regulate sumoylation at the target level is through target phosphorylation (Figure 2). As will become evident from the examples below, phosphorylation may serve both as a positive and a negative signal for sumoylation. An example for negative regulation is the sumoylation of AP-1 transcription factors c-Jun and c-Fos, which is reduced after phosphorylation of specific residues (Ser 63 and 73 for c-Jun and Thr 232 for c-Fos) by JNK (in the case of c-Jun) and by an unidentified, Ras activated, kinase in the case of c-Fos [25,26]. Consistent with the finding that sumoylation inhibits their transcriptional activity, phosphorylation of these residues is known to activate c-Jun and c-Fos dependent transcription. Along the same line, phosphorylation of p53 at Ser 20, induced by DNA damaging agents, strongly reduces its sumoylation [27]. ELK-1 phosphorylation upon activation of the MAPK pathway was also shown to inhibit its sumoylation [28]. Whether phosphorylation serves to recruit isopeptidases, blocks access of Ubc9 or E3 ligases, or prevents modification by other mechanisms is currently unknown.

In other cases, phosphorylation can enhance sumoylation. This was first demonstrated for the Heat Shock Factor HSF1. Its phosphorylation on Ser 303, induced after heat shock, is required for efficient sumoylation on the nearby Lys 298 [29]. A S303E mutant is more efficiently sumoylated than wt even in vitro, suggesting that the underlying mechanism is enhanced recruitment of the E2 conjugating enzyme Ubc9 E3 (ligases were absent in the assay). Whether this involves a conformational change or whether the acidic residue contributes directly to Ubc9 association awaits further analysis. Importantly, these findings could be generalized: The sequence motif, ΨKxExxSP, which is now referred to as Phosphorylation Dependent Sumoylation Motif (PDSM) [30], is present in various proteins, predominantly transcriptional regulators. For several of these (HSF4, GATA-1, MEF2, SNIP-1 and ERRγ) the authors demonstrated that serine phosphorylation in the PDSM indeed enhances their sumoylation [30]. Phosphorylation dependent sumoylation of MEF2 family members was also reported by other groups [31,32].

Regulated MEF2 sumoylation seems to actually involve an added level of complexity: Zhao and coworkers [33] had already observed that MEF2D can be sumoylated or acetylated on the same lysine residue. A report by Shalizi et al., [34] now brings together phosphorylation, acetylation and sumoylation of MEF2A: Calcineurin dependent dephosphorylation of Ser 408 in MEF2A, which is located in the PDSM, was shown to promote a switch from sumoylation to acetylation at lysine 403. Consequently, sumoylation of MEF2 is inhibited not only by the lack of the phosphate in the PDSM, but also by direct competition of acetylation and sumoylation on the same lysine residue.

The concept that sumoylation and acetylation are two competing modifications for the same lysine residue in some SUMO targets is actually not new – it has been first noted for the transcription factor Sp3 [35], and was also shown for the transcriptional co-activator p300, whose deacetylation by SIRT1 is required for its efficient sumoylation [36]. Whether sumoylation serves to inhibit acetylation, whether acetylation serves to prevent sumoylation, or whether the sumoylated, acetylated and non-modified species serve independent functions is one of many interesting questions that arise from these studies. One particularly interesting question is whether lysine acetylation frequently overlaps with SUMO acceptor sites.

Lysine residues are obviously not only targets for sumoylation and acetylation, but can, e.g., also be ubiquitinylated. This brings us to another type of competing modifications – ubiquitinylation and sumoylation (Figure 2). The first example is Iκ-Bα that can be poly-ubiquitinylated or mono-sumoylated on lysine 21 [37]. While poly-ubiquitinylation and subsequent degradation by the proteasome requires signal-induced phosphorylation of Iκ-Bα, sumoylation takes place in the absense of phosphorylation. It has been suggested that SUMO serves to protect Iκ-Bα from degradation, however only a very small fraction of Iκ-Bα is sumoylated at steady state. More recently, it has been shown that NEMO, the Iκ-B kinase (IKK) regulator, is also subject to both modifications on the same lysine [38]. Here, sumoylation/desumoylation and ubiquitinylation may be independent, but consecutive, events in the activation pathway [39]. Finally, *S.c. and Xenopus laevis *PCNA were shown to be mono-ubiquitinylated, poly-ubiquitinylated (K63 chains) or sumoylated on lysine 164. Different modifications are induced by different cues (e.g., cell cycle position or DNA damage), and have distinct as well as cooperative functions in DNA repair and replication (reviewed in [40]). In conclusion, quantitative and stable modification of a given target with SUMO or ubiquitin would obviously prevent modification with the other moiety, and either modifier could hence serve as an antagonist of the other. However, from the currently known data it seems more likely that both types of modifications are regulated independent of each other and serve different roles (reviewed in [41]).

The mechanisms discussed above all involve posttranslational modifications of the target protein. Similar mechanisms can obviously be envisioned at the level of target specific E3 ligases or isopeptidases. Modification of these enzymes may well alter their ability to interact with specific target proteins. Finally, many enzymes in the SUMO pathway have restricted intracellular localisations (reviewed in [7]). Hence sumoylation/desumoylation of specific targets requires targeting to those sites. This has, e.g., been shown for SP100, whose sumoylation requires nuclear targeting [43]. Consequently, any event that changes target or enzyme position within the cell may influence the extent of sumoylation for a specific target. An intriguing example is the relocalisation of the *S. cerevisiae *E3 ligase Siz1 from the nucleus to the bud neck in mitosis, which results in septin sumoylation [42].

## Regulation of the SUMO conjugating and deconjugating enzymes

**Figure 3:**
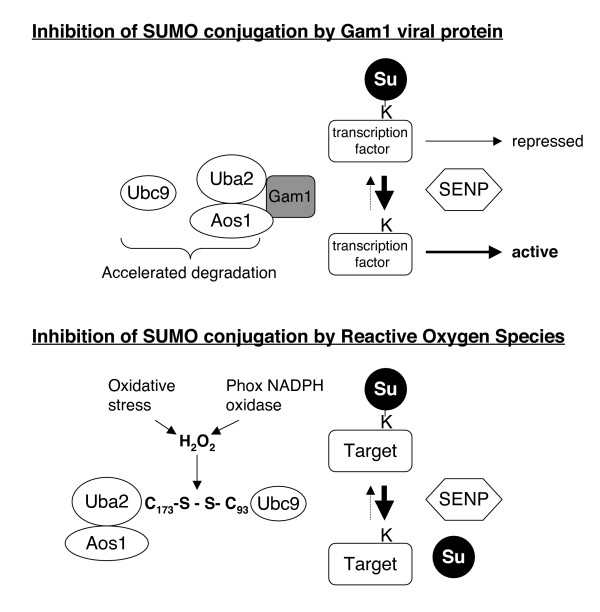
**Global regulation of the conjugating machinery**. A) The Gam1 protein from CELO adenovirus reduces E1 and E2 enzyme levels [47]. The resulting global desumoylation correlates with increased transcriptional activity, which is due, at least in part, to desumoylation of transcription factors such as Sp3. B) H_2_O_2 _inhibits SUMO conjugation by inducing the formation of a disulfide bond between the catalytic cysteines of the E1 and E2 enzymes. Depending on the source and dose of H_2_O_2 _(applied exogenously or produced endogenously) this results in local desumoylation of specific proteins or global desumoylation [52].

Another, more global, way to regulate sumoylation is by directly targeting the basic conjugation or deconjugation machineries (Figure 3). As in many other cases, viruses understood this was an efficient way to regulate the sumoylation pathway for their own sake. Some viral proteins from cytomegalovirus (CMV) Herpes Simplex Virus (HSV) or adenoviruses were shown to repress sumoylation of specific proteins [44-46], although the underlying mechanisms were not characterized. A better-documented case of viral protein interfering with the SUMO conjugation pathway is the Gam1 protein from the CELO adenovirus [47]. It was shown that expression of the Gam1 protein leads to a drastic decrease in cellular SUMO conjugates. This loss of sumoylated proteins correlated with the disappearance of SUMO E1 and E2, suggesting that Gam1 could target the conjugating enzymes to degradation. What is the benefit of targeting the SUMO pathway for virus propagation? As previously outlined, sumoylation globally represses signal transduction and gene expression. Therefore, by preventing sumoylation, the CELO adenovirus may globally enhance the transcription capacity of the cell, facilitating its own propagation. This seems indeed to be the case, as Gam1 expression enhanced transcriptional activity via its effect on the SUMO pathway [47]. Gam1 can interact directly with the SUMO E1 activating enzyme, but how this triggers its disappearance is presently unknown. An attractive hypothesis is the recruitment of the ubiquitinylation machinery.

The SUMO conjugation/deconjugation pathway is also globally regulated by stresses. Various stresses, including heat shock, osmotic and high oxidative stress (H_2_O_2_) enhance global sumoylation both in mammals [48], plants[49] and yeasts [50]. In mammals, the increase of conjugates was largely observed for SUMO-2/3 but not for SUMO-1. The apparent difference in stress response between SUMO-1 and SUMO-2/3 may point to some interesting difference in regulation, but could also have a rather trivial explanation: there is a large pool of free SUMO-2 and -3 but limited SUMO-1 under normal conditions [48]. A significant increase of SUMO-2/3 conjugation has also been observed in HEK293 cells exposed to the lipid oxidation-derived electrophile 4-hydroxynonenal, which is an ubiquitous product of lipid oxidation associated with oxidative stress [51].

In the case of oxidative stress in mammalian cells, the situation is more complex than it first appeared. Indeed, increased SUMO conjugation was observed only in conditions of extreme oxidative stress (100 mM H_2_O_2_), which does not occur under physiological or even pathophysiological conditions in vivo. At more relevant concentrations (1 mM and below), H_2_O_2 _has opposite effects on SUMO conjugation, leading to almost complete loss of SUMO-1, -2 and -3 conjugates. Mechanistically, this is due to direct inhibition of SUMO conjugation by induction of a reversible disulfide bridge between the catalytic cysteines of the E1 subunit Uba2 and the E2 Ubc9. Under those conditions, SUMO isopeptidases, which also have a cysteine in their active site, are almost not affected [52]. As a consequence, this shifts the equilibrium in the sumoylation-desumoylation cycle towards unconjugated species (increased sumoylation at the unphysiological dose of 100 mM H_2_O_2 _can perhaps be explained by simultaneous inhibition of modifying and demodifying isopeptidases enzymes). What is the benefit for the cell to block sumoylation in case of oxidative stress? Again, this might help to activate transcription of genes involved in the return to normal redox conditions. Supporting this explanation, AP-1 transcription factors c-Fos and c-Jun are desumoylated within a few minutes after H_2_O_2 _addition to the cells. Considering that (i) sumoylation represses their activity [25,26] and (ii) they play a key role in the transcriptional activation of numerous anti-oxidant proteins [53], their rapid desumoylation might play a key role in the early response to oxidative stress.

The sensitivity of SUMO pathway enzymes towards reversible inactivation by oxidation raises the question of whether this mechanism is also important for physiological processes that involve redox signaling. Unlike "oxidative stress" which is characterized by a macroscopic shift in cellular redox potential, redox signaling involves reversible, locally and chemically defined, oxidations of key enzymes in signaling pathways [54,55]. This involves, e.g., peptide growth factor induced activation of membrane associated NADPH oxidases that produce H_2_O_2_. NADPH-Oxidase complexes have been found in most cell types. Transient accumulation of hydrogen peroxide is known to participate in receptor signaling by, e.g., reversibly oxidizing the essential cysteine residues of protein tyrosine phosphatases and the lipid phosphatase PTEN [55].

Highest levels of endogenous H_2_O_2 _production are known to occur in phagocytic cells. Their activation by endotoxins (such as LPS) or inflammatory mediators (such as TNF, Interferon or Phorbol esters) is responsible for the so-called respiratory burst, which consists in the production of ROS by a specific NADPH oxidase complex called Phox [54]. While it was long thought that this ROS production was only aimed at killing the engulfed pathogen, it is now known that these ROS also play a key role as second messengers in AP-1 and NF-κB signaling pathways [56]. At least in this system, or more precisely in a macrophage cell line activated by PMA and zymosan, endogenously produced H_2_O_2 _is sufficient to reversibly inactivate a significant fraction of cytoplasmic SUMO E1 and E2 enzymes. While the global pattern of SUMO conjugates remains unchanged, some demodification of unknown proteins has been observed [52]. One attractive hypothesis is that signal induced desumoylation of specific proteins in the immediate vicinity of NADPH oxidases, both in macrophages and other cell types, may contribute to activation of signal transduction and subsequent cellular responses.

## Conclusion

Considering the enormous number of sumoylated proteins that are modified in temporally and spatially controlled manner, the number of known enzymes is surprisingly limited. Nevertheless, in vitro, these enzymes are sufficient to modify many proteins. To account for the high specificity observed in vivo, we therefore have to assume the existence of many regulatory mechanisms. As outlined in this review, we are now beginning to understand how sumoylation can be regulated -mechanisms such as phosphorylation of selected targets and enzymes, antagonistic modifications and local inactivation by oxidation appear to contribute at the level of individual targets and in some scenarios globally. Different aspects of these and other regulatory mechanisms will undoubtedly be the focus of many exciting contributions over the next few years.
